# Absetzen anfallssuppressiver Medikamente bei Patient*innen mit Epilepsie

**DOI:** 10.1007/s00115-024-01708-3

**Published:** 2024-07-18

**Authors:** Maria Ilyas-Feldmann, Luise Graf, Thea Hüsing, Jakob Dörrfuß, Martin Holtkamp

**Affiliations:** Epilepsie-Zentrum Berlin-Brandenburg, Klinik für Neurologie mit Experimenteller Neurologie, Charité – Universitätsmedizin Berlin, Charitéplatz 1, 10117 Berlin, Deutschland

**Keywords:** Anfallsfreiheit, Anfallsrezidiv, Gemeinsame Entscheidungsfindung, Nebenwirkungen, Seizure freedom, Seizure recurrence, Shared decision-making, Side effects

## Abstract

**Hintergrund:**

Etwa zwei Drittel der Patient*innen mit Epilepsie werden unter der Einnahme anfallssuppressiver Medikamente (ASM) anfallsfrei. Eine zentrale Frage ist, ob und wann ASM wieder abgesetzt werden können.

**Ziel der Arbeit:**

Überblick zum aktuellen Kenntnisstand über Risiken und Nutzen des Absetzens von ASM.

**Methoden:**

Zusammenfassung der aktuellen Literatur, Diskussion der Datenlage und Ableitung von Therapieempfehlungen.

**Ergebnisse:**

Das Risiko für Anfallsrezidive nach dem Absetzen von ASM ist mit 40–50 % ungefähr doppelt so hoch wie unter der weiteren Einnahme von ASM. Leitlinien empfehlen, das Absetzen von ASM frühestens nach 2‑jähriger Anfallsfreiheit zu erwägen. Prädiktive Faktoren für ein Anfallsrezidiv nach dem Absetzen von ASM umfassen eine längere Dauer der Epilepsie und eine höhere Anzahl epileptischer Anfälle bis zur klinischen Remission, ein kürzeres anfallsfreies Intervall bis zum Absetzen, ein höheres Alter bei Erstmanifestation, eine Entwicklungsverzögerung bzw. ein IQ < 70, Fieberkrämpfe in der Kindheit, das Nichtvorliegen eines selbstlimitierenden Epilepsiesyndroms und der Nachweis epilepsietypischer Muster im EEG. Mithilfe einer webbasierten Prognosesoftware kann das individuelle Risiko eines Anfallsrezidivs nach dem Absetzen von ASM abgeschätzt werden.

**Schlussfolgerungen:**

Ein Absetzen von ASM sollte frühestens nach 2 Jahren Anfallsfreiheit in einer gemeinsamen Entscheidungsfindung von Ärzt*innen und Patient*innen unter Abwägung von Nutzen und Risiken besprochen werden. Das Risiko eines erneuten Anfalls wird durch eine Reihe klinischer Variablen beeinflusst. Psychosoziale Aspekte wie Fahreignung und die berufliche Situation müssen ebenso berücksichtigt werden wie individuelle Ängste und Sorgen der Patient*innen vor einem Anfallsrezidiv oder der dauerhaften Einnahme von ASM.

## Abstract (en)

**Background:**

Approximately two thirds of patients with epilepsy become seizure-free with antiseizure medication (ASM). A central question is whether and when ASM can be discontinued.

**Objective:**

To present an overview of the current knowledge about risks and benefits of discontinuation of ASM.

**Material and methods:**

Review of the current literature, discussion of data on and recommendations for discontinuation of ASM.

**Results:**

The risk of seizure recurrence after discontinuation of ASM is approximately 40–50% and thus twice as high as continuing with ASM. Guidelines recommend considering discontinuation of ASM at earliest after a seizure-free period of 2 years. Predictive variables for seizure recurrence after stopping ASM include longer duration of epilepsy and higher number of seizures until remission, a shorter seizure-free interval until stopping ASM, older age at epilepsy onset, developmental delay or IQ < 70, febrile seizures in childhood, absence of a self-limiting epilepsy syndrome, and evidence of epileptiform activity in the electroencephalograph (EEG). The individual risk of seizure recurrence after stopping ASM can be estimated using an online prediction tool.

**Conclusion:**

Discontinuation of ASM should be discussed with patients at the earliest after 2 years of seizure freedom in a shared decision-making process weighing up the risks and benefits. The risk of a seizure recurrence depends on a number of clinical variables. Psychosocial aspects, such as impact on driving and occupational issues must be taken into consideration as well as individual fears and concerns of patients about seizure recurrence or the long-term use of ASM.

## Hintergrund

Epilepsien gehören zu den häufigsten schweren neurologischen Erkrankungen. Der Grundpfeiler der Behandlung sind anfallssuppressive Medikamente (ASM). Etwa zwei von drei Patient*innen werden mit ASM anfallsfrei [1, 2]. Auch wenn mithilfe von ASM eine langjährige Anfallsfreiheit erreicht wird, spricht man nicht von einer „Heilung“ der Erkrankung. Eine Epilepsie gilt als überwunden (oder „ausgestanden“, „abgeklungen“ – im englischen Original „resolved“), wenn Patient*innen 10 Jahre anfallsfrei waren und mindestens in den letzten 5 Jahre kein ASM eingenommen haben [3, 4]. Eine bevölkerungsbasierte Studie der Mayo Clinic in Rochester, USA, zeigte, dass sich 20 Jahre nach der Erstdiagnose einer Epilepsie 70 % der Patient*innen in Remission befanden (hier definiert als mindestens 5 Jahre anfallsfrei), etwa die Hälfte dieser Patient*innen nahm seit mindestens 5 Jahren kein ASM mehr ein [5]. Wesentlich für anfallsfreie Patient*innen ist die Frage, ob und wann ein ASM abgesetzt werden kann. Das Absetzen eines ASM sollte nach einer sorgfältigen Nutzen-Risiko-Abwägung gemeinsam von Ärzt*in und Patient*in entschieden werden [4, 6]. Dabei müssen individualisiert die Wünsche, Erwartungen und Sorgen von Patient*innen sowie deren aktuelle berufliche und private Lebenssituation berücksichtigt werden.

Im Folgenden geben wir einen Überblick über aktuelle Studien zum Rezidivrisiko für Anfälle nach dem Absetzen eines ASM bei erwachsenen Patient*innen mit Epilepsie. Wir werden nicht auf das Absetzen von ASM nach einem akut-symptomatischen Anfall oder nach einem epilepsiechirurgischen Eingriff eingehen.

## Nutzen und Risiken des Absetzens von ASM

Der Nutzen des Absetzens eines ASM kann in einem Wegfall etwaiger Nebenwirkungen und Wechselwirkungen [7] und somit in der Verbesserung der Lebensqualität bestehen (Tab. 1). Nebenwirkungen bei der Langzeitbehandlung mit ASM können neuropsychologische und -psychiatrische (kognitive Beeinträchtigung, Depressionen, Reizbarkeit; [8]), neurologische (Schwindel, Doppelbilder, Tremor) als auch allgemeine, z. B. internistische (Übelkeit, Leberschädigungen, Herz-Kreislauf-Erkrankungen; [9–11]) oder teratogene Aspekte umfassen [12, 13]. Die Häufigkeit angegebener Nebenwirkungen variiert in Abhängigkeit von der Art ihrer Erhebung. Spontan berichten 10–40 % der Patient*innen über Nebenwirkungen [14]. Unter Verwendung strukturierter Screeningfragebögen geben 60–90 % der Patient*innen Nebenwirkungen ihrer ASM an [15, 16]. Oftmals werden Nebenwirkungen von Patient*innen subjektiv nicht wahrgenommen, und sie werden erst deutlich, wenn das ASM abgesetzt wurde. Dies gilt insbesondere für kognitive Beeinträchtigungen durch ASM [17]. ASM haben zudem potenzielle Wechselwirkungen mit anderen Medikamenten. Vor allem die Interaktion von Substanzen wie Lamotrigin mit hormonellen Kontrazeptiva ist von hoher klinischer Relevanz [18]. Darüber hinaus sind einige ASM, wie Carbamazepin und Phenytoin, Induktoren bestimmter Zytochrom-P450-Isoenzyme. Sie können z. B. die Wirksamkeit oraler Antikoagulanzien sowie antiviraler oder antibiotischer Medikamente beeinflussen [19].

**Tab. 1:** Nutzen und Risiken des Absetzens von Anfallssuppressiva

Nutzen	Risiken
Wegfall etwaiger Nebenwirkungen	Anfallsrezidiv: Verletzungsrisiko Psychosoziale Folgen (Berufstätigkeit, Partnerschaft, Fahreignung) Ängste und Unsicherheit der Patient*innen
Wegfall etwaiger Wechselwirkungen	
Verbesserung der Lebensqualität und Reduktion von Stigmatisierung	
Kostenreduktion im Gesundheitssystem und für Patient*innen	

Darüber hinaus kann die langjährige Einnahme von ASM die Lebensqualität der Patient*innen reduzieren [20], und das Absetzen kann die Lebensqualität verbessern [21]. Damit einhergehend kann sich auch eine von Patient*innen empfundene Stigmatisierung [22] wegen der täglichen Einnahme von ASM durch deren Absetzen bessern [23, 24]. In spezifischen Fällen können durch das erfolgreiche Absetzen von ASM und nach mehrjähriger anfallsfreier Beobachtungszeit auch berufliche Einschränkungen wegfallen. So schließt die Einnahme von ASM die Kraftfahreignung für Fahrzeuge der Gruppe 2 (LKW und Fahrgastbeförderung) aus.

Ein weiterer Nutzen des Absetzens von ASM ist die Reduktion von Kosten für das Gesundheitssystem im Allgemeinen sowie für die Patient*innen bei Zuzahlungspflicht im Besonderen. Die direkten Kosten von Epilepsien für die Gesellschaft und das Gesundheitssystem betrugen schon vor 10 Jahren ca. 1700 € pro Patient*in und Jahr, mehr als die Hälfte der Kosten entfällt auf ASM [25]. Es gibt aber aktuell keine Untersuchung, die Einsparungen durch unnötige Ausgaben für ASM den Kosten eines Anfallsrezidivs nach Absetzen von ASM gegenüberstellt.

Den dargestellten Nutzen stehen Risiken eines Absetzversuchs gegenüber, die durch das Auftreten eines erneuten epileptischen Anfalls und die damit verbundenen Folgen entstehen. Neben den unmittelbaren Risiken jedes epileptischen Anfalls (u. a. Sturz mit Verletzung) besteht das (recht geringe) Risiko, dass sich ein Anfallsrezidiv ohne ASM als Status epilepticus manifestiert oder dass es im Zuge eines fokal zu bilateralen oder generalisierten tonisch-klonischen Anfalls zu einem plötzlichen, unerwarteten Tod bei Epilepsie („sudden unexpected death in epilepsy“ [SUDEP]) kommt.

Darüber hinaus besteht die Gefahr, dass Patient*innen mit einem Anfallsrezidiv nach dem Absetzen von ASM trotz erneuter Einnahme nicht wieder anfallsfrei werden. Einem systematischen Review mit 14 Studien zufolge konnte bei 19 % der Patient*innen in dieser Konstellation keine erneute Anfallskontrolle erreicht werden [26]. Bei der Interpretation dieser Zahl muss aber berücksichtigt werden, dass auch langjährig anfallsfreie Patient*innen unter kontinuierlicher Einnahme von ASM spontane Anfallsrezidive haben können und dann in 25 % der Fälle nicht wieder anfallsfrei werden [27].

Das Wiederauftreten eines Anfalls nach dem Absetzen von ASM kann relevante psychosoziale Auswirkungen haben [22]. Neben Ängsten und Unsicherheiten bei den Patient*innen führt ein Anfallsrezidiv zu einem vorübergehenden Verlust der Fahreignung, zudem bestehen mögliche Einschränkungen der beruflichen Einsatzfähigkeit [28].

## Risiko für ein Anfallsrezidiv nach Absetzen von ASM

Die Evidenzlage hinsichtlich des Risikos für ein Anfallsrezidiv nach dem Absetzen von ASM im Vergleich zur Fortführung von ASM ist bei erwachsenen Patient*innen mit Epilepsie gering. Es gibt zwei randomisierte kontrollierte Studien, in beiden Studien war ein anfallsfreies Intervall vor dem Absetzen des ASM in Monotherapie von mindestens 2 Jahren gefordert. Die größere der beiden Studien ist vom Medical Research Council in Großbritannien durchgeführt worden und war eine randomisierte kontrollierte, aber nicht doppelt-verblindete Studie [29]. Von gut 1000 Patient*innen mit Epilepsie wurde bei der Hälfte über einen Zeitraum von mindestens 6 Monaten das ASM ausgeschlichen, die andere Hälfte wurde unverändert mit einem ASM weiterbehandelt. Der Anteil der Patient*innen mit einem Anfallsrezidiv betrug 24 Monate nach Absetzen des ASM 41 %, unter Weitereinnahme des ASM lag das Risiko bei 22 %. Die zweite und einzige randomisierte kontrollierte, doppelt-verblindete Studie wurde in Norwegen mit insgesamt 160 Patient*innen durchgeführt [17]. Bei den 79 Patient*innen, bei denen das ASM abgesetzt wurde, betrug die Rate an Anfallsrezidiven nach 12 Monaten 15 %, unter Fortführung des ASM (*n* = 81) lag diese bei 7 % [17]. Der Unterschied zwischen den beiden Gruppen 12 Monate nach Absetzen der ASM war jedoch statistisch nicht signifikant (RR 2,46; 95 %-KI: 0,85–7,08; *p* = 0,095). In einer Metaanalyse von zehn prospektiven und retrospektiven Studien mit mehr als 1700 Patient*innen hatten 46 % nach einem medianen Follow-up von 5 Jahren nach Absetzen der ASM ein Anfallsrezidiv [30].

Zusammenfassend ist das Risiko für das Wiederauftreten eines epileptischen Anfalls nach Absetzen der ASM ungefähr doppelt so hoch wie unter Fortführung der Medikation, es liegt, unabhängig von der Epilepsieart und Ätiologie, innerhalb der ersten 5 Jahre nach Absetzen bei etwa 40–50 %.

Basierend auf den erwähnten Studien kann das Absetzen eines ASM nach einer Mindestdauer von 2 Jahren Anfallsfreiheit erwogen werden [4, 31, 32]. Das Risiko eines erneuten Anfalls nach dem Absetzen des ASM sinkt, je länger Patient*innen anfallsfrei waren [33–35]. Eine Studie konnte zeigen, dass Patient*innen, die mindestens 5 Jahre anfallsfrei waren, nach dem Absetzen des ASM ein gleich hohes Risiko für ein Anfallsrezidiv hatten wie Patient*innen, die das ASM weiter einnahmen (Hazard Ratio 1,362; 95 %-KI 0,634–2,926; *p* = 0,428; [33]).

## Risikofaktoren für ein Anfallsrezidiv nach Absetzen von ASM

In der oben erwähnten Metaanalyse wurden acht unabhängige Risikofaktoren für ein Anfallsrezidiv identifiziert (Tab. 2; [30]). Die Autor*innen entwickelten hieraus eine frei zugängliche webbasierte Prognosesoftware (http://epilepsypredictiontools.info) (UMC Utrecht, The Netherlands). Basierend auf den Prädiktoren der hier genannten Metaanalyse kann das individuelle Risiko für Anfallsrezidive 2 und 5 Jahre nach Absetzen abgeschätzt werden.

**Tab. 2:** Unabhängige Faktoren für Anfallsrezidive nach dem Absetzen von Anfallssuppressiva

Längere Dauer der Epilepsie bis zur klinischen Remission
Höhere Anzahl epileptischer Anfälle bis zur klinischen Remission
Kürzeres anfallsfreies Intervall bis zum Absetzen
Höheres Lebensalter bei Krankheitsbeginn
Entwicklungsverzögerung bzw. IQ<70
Fieberkrämpfe in der Kindheit
Nichtvorliegen eines selbstlimitierenden Epilepsiesyndroms
Nachweis epilepsietypischer Muster im EEG

Bei der Abschätzung des Risikos für ein Anfallsrezidiv nach dem Absetzen des ASM stellen auch die Epilepsieart und das Epilepsiesyndrom relevante Faktoren dar. So ist bei in der Kindheit beginnenden genetischen Epilepsien, wie der Absence-Epilepsie oder der Rolando-Epilepsie, die Chance sehr hoch, dass es nach langjähriger Anfallsfreiheit auch nach dem Absetzen einer ASM zu keinem erneuten Anfall kommt [36–38]. Dagegen ist die Wahrscheinlichkeit für ein Anfallsrezidiv nach dem Absetzen eines ASM bei Patient*innen mit einer fokalen Epilepsie mit struktureller Läsion im MRT, insbesondere einer Hippokampussklerose [37, 39], oder bei Patient*innen mit einer juvenilen myoklonischen Epilepsie (JME), dem häufigsten Syndrom innerhalb der genetisch generalisierten Epilepsien, hoch. In einer Studie mit 84 Patient*innen mit fokaler Epilepsie hatten nach dem Absetzen des ASM 38 % der Patient*innen mit einem normalen MRT und 72 % der Patient*innen mit Hippokampusatrophie im MRT ein Anfallsrezidiv [39]. Bei Patient*innen mit einer JME galt über Jahrzehnte das Dogma, dass bei sehr hohem Rezidivrisiko ASM lebenslang eingenommen werden müssen. Eine Reihe von Studien, hat dieses Paradigma aber in den letzten Jahren relativiert, bis zu 25 % der Patient*innen waren nach dem Absetzen des ASM mindestens 5 Jahre anfallsfrei [40–42]. In einer Metaanalyse mit 2518 Patient*innen mit JME wurde bei 368 Patient*innen das ASM abgesetzt. Von diesen Patient*innen hatten 73 % (95 %-KI 67–78 %) innerhalb der nächsten 5 Jahre ein Anfallsrezidiv [43]. Basierend auf diesen Daten haben die Autor*innen auch für Patient*innen mit JME Nomogramme erstellt und dieses Epilepsiesyndrom als Subgruppe in die oben erwähnte Prognosesoftware zur Abschätzung des individuellen Risikos für ein Anfallsrezidiv unter Berücksichtigung prädiktiver klinischer Variablen integriert (http://epilepsypredictiontools.info) (UMC Utrecht, The Netherlands).

## Praktisches Vorgehen beim Absetzen von ASM

Patient*innen sollten darüber aufgeklärt werden, dass ASM nur nach ärztlicher Rücksprache abgesetzt werden sollten, um das Risiko für einen Status epilepticus oder Entzugsanfälle zu minimieren [44]. Belastbare Daten für Erwachsene zur Dauer der Dosisreduktion bis zum Absetzen des ASM fehlen bislang. In neuropädiatrischen Studien fand sich im Vergleich von schnellem (innerhalb von 4 bis 6 Wochen) und langsamem (innerhalb von 9 bis 12 Monaten) Absetzen des ASM kein Unterschied in der Rate von Anfallsrezidiven [45]. In der einzigen Studie mit 48 erwachsenen Patient*innen mit fokaler oder generalisierter Epilepsie, der Studie „RApid versus SLOW withdrawal (RASLOW)“ [46], wurde bei 25 Patient*innen das ASM langsam über 160 Tage (Reduktion der ASM-Dosis um 20 % alle 40 Tage) und bei 23 Patient*innen schneller über 60 Tage (Reduktion der ASM-Dosis um 20 % alle 15 Tage) abgesetzt. Nach einem medianen Beobachtungszeitraum von knapp 1 Jahr kam es bei drei Patient*innen in der Gruppe mit langsamem Absetzen und bei einer Patient*in in der Gruppe mit schnellem Absetzen zu einem Anfallsrezidiv. Keine*r der Patient*innen entwickelte einen Status epilepticus, anfallsbedingte Verletzungen oder starb.

Einige ASM weisen jedoch entweder wegen Gewöhnung (GABAerge Substanzen wie Benzodiazepine, Phenobarbital, Primidon) oder aufgrund ihres Wirkmechanismus (Vigabatrin als irreversibler Hemmstoff der GABA-Transaminase) bei schneller Reduktion der Dosis oder sofortigem Absetzen ein erhöhtes Risiko für Entzugsanfälle auf. Bei diesen ASM sollte eine langsame Reduktion der Dosis bis zur Beendigung der Therapie über einige Monate hinweg erfolgen [44].

Patient*innen müssen darüber aufgeklärt werden, dass für die Dauer des Absetzens und für 3 Monate nach der letzten Einnahme des zuvor in Monotherapie eingenommenen ASM keine Fahreignung für Kraftfahrzeuge besteht. Bei langsamem Absetzen verlängert sich daher die Dauer der fehlenden Fahreignung.

Einer der unabhängigen Risikofaktoren für ein Anfallsrezidiv nach dem Absetzen von ASM ist der Nachweis interiktaler epileptiformer Muster im EEG noch unter der Einnahme von ASM [30], dies gilt insbesondere für Patient*innen mit genetisch generalisierter Epilepsie. Bei dieser Epilepsieart ist daher ein Routine-EEG vor dem etwaigen Absetzen des ASM sinnvoll. Ein Langzeit-EEG über mindestens 24 h kann die Sensitivität für interiktale epileptiforme Muster erhöhen, ist aber wegen der limitierten Ressourcen in der Mehrzahl der Fälle nicht umsetzbar [47]. Der exakte Stellenwert etwaiger neu aufgetretener epileptiformer Muster im EEG während oder nach dem Absetzen von ASM ist unklar [48–50].

Nach Beendigung eines ASM sollten Patient*innen für mindestens 2 Jahre von ihren behandelnden Neurolog*innen regelmäßig gesehen werden [31], um nach klinisch diskreten Anfällen wie fokalen bewusst erlebten nichtmotorischen Anfällen (früher: Aura) und Myoklonien bei genetisch generalisierten Epilepsien zu fragen. Möglicherweise interpretieren die Patient*innen diese Symptome nicht als epileptische Anfälle [47].

## Gründe aus Sicht von Patient*innen und Ärzt*innen, ein ASM nicht abzusetzen

Patient*innen und Ärzt*innen sind auch nach mehrjähriger Anfallsfreiheit oft gegenüber dem Absetzen von ASM ambivalent. In einer kürzlich veröffentlichten retrospektiven Arbeit konnten wir zeigen, dass das Nichtabsetzen von ASM in Monotherapie mit früheren fokal zu bilateralen oder generalisierten tonisch-klonischen Anfällen, einer höheren Dosis der ASM, vorangegangenen spontanen Anfallsrezidiven nach langjähriger Anfallsfreiheit sowie fehlgeschlagenen vorherigen Absetz- oder Dosisreduktionsversuchen unabhängig assoziiert war [51].

Zwischen 55–76 % der Patient*innen, denen ärztlicherseits ein Absetzen ihres ASM empfohlen wurde, entschieden sich in zwei Studien dagegen und bevorzugen eine weitere Einnahme des ASM [28, 51]. Gründe der Patient*innen gegen ein Absetzen von ASM trotz Anfallsfreiheit umfassen das Gefühl, gut auf das ASM eingestellt zu sein, die Sorge vor einem Anfallsrezidiv mit der Konsequenz der – wenn auch nur vorübergehenden – fehlenden Fahreignung und ggf. des Verlusts des Arbeitsplatzes, einen vorherigen Absetzversuch mit nachfolgendem Anfallsrezidiv und das frühere Auftreten pharmakologisch nicht kontrollierter epileptischer Anfälle [28].

Auf der anderen Seite bestehen auch auf professioneller Seite Unsicherheiten, Patient*innen hinsichtlich des Absetzens eines ASM adäquat zu beraten, und ein Absetzversuch wird von ärztlicher Seite eher selten thematisiert. In einer Studie aus Norwegen wurde nur bei 60 von 186 (32 %) Patient*innen, die seit mindestens 5 Jahren anfallsfrei waren, über ein Absetzen des ASM gesprochen [52]. In zwei weiteren größeren retrospektiven Studien war nur bei der Hälfte der Patient*innen ein gemeinsames Gespräch mit Ärzt*innen über das Absetzen des ASM dokumentiert [51, 53]. Letztlich wurde jedoch nur bei 98 der 463 (21 %) eingeschlossenen Patient*innen das Absetzen des ASM konkret geplant [53].

## Zusammenfassung und Fazit für die Praxis

Ob und wann bei anfallsfreien Patient*innen mit Epilepsie die regelmäßige Einnahme von anfallssuppressiven Medikamenten (ASM) beendet werden kann, ist eine komplexe und vielschichtige Frage. Das Risiko eines Anfallsrezidivs ist nach dem Absetzen von ASM etwa doppelt so hoch wie unter der Fortführung der Therapie und beträgt nach 5 Jahren etwa 40–50 %. Die Wahrscheinlichkeit von Anfallsfreiheit nach dem Absetzen von ASM hängt von einer Reihe von Faktoren ab. Bei erwachsenen Patient*innen mit unklassifizierter oder fokaler Epilepsie sowie selbstlimitierenden Epilepsiesyndromen, unauffälligem EEG und MRT, wenigen Anfällen und kurzer Dauer der Epilepsie bis zum Erreichen von Anfallsfreiheit, einem IQ > 70 und einem normalen neurologischen Untersuchungsbefund besteht nach dem Absetzen der ASM eine höhere Chance auf Anfallsfreiheit. Nach den Empfehlungen der aktuellen Leitlinie *Erster epileptischer Anfall und Epilepsien im Erwachsenalter* der Deutschen Gesellschaft für Neurologie kann bei mindestens zweijähriger Anfallsfreiheit in einer gemeinsamen Entscheidungsfindung von Ärzt*in und Patient*in (im Sinne des „shared decision-making“) ein Absetzen des ASM desto eher erwogen werden, je günstiger die Konstellation bezüglich der genannten Prädiktoren für weitere Anfallsfreiheit ist [4]. Zudem sollen laut Leitlinie die Patient*innen darüber aufgeklärt werden, dass bei der gemeinsamen Entscheidungsfindung die wahrscheinlichen Konsequenzen eines möglichen Rezidivs bzgl. Fahreignung und beruflicher Tätigkeit dem möglichen Nutzen der Beendigung der ASM-Therapie gegenüberzustellen sind.
